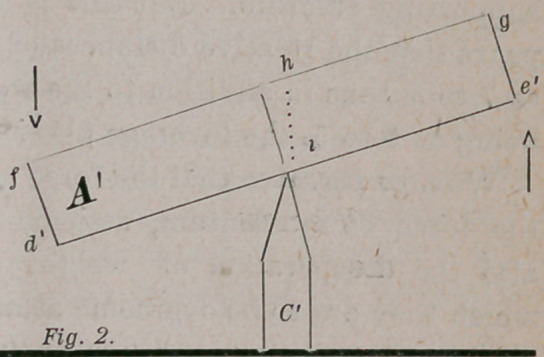# Equilibrium, the Controlling Force in Nature

**Published:** 1887-08

**Authors:** John Franklin Clark


### EQUILIBRIUM THE CONTROLLING FORCE IN NATURE.

BY JOHN FRANKLIN CLARK.

There are three modes of force that seemingly, are universal in their operative effects, which are designated by the terms equilibrium, vibration and gravitation, and of these three modes of force, which, possibly, it might be justifiable to designate as its fundamental modes, it is proposed to demonstrate that equilibrium is the dominating mode, ever operating to bring ponderable bodies to a state of equipoise.

It is stated, as a law of physics, that a body in a state of rest can only be put in motion, by an applied force sufficient to overcome its inertia, and that having been so put in motion, it can only be brought to a state of rest, by a resisting medium or force, sufficient to overcome its momentum.

It is also stated as a law of physics, that the gravitative force increases as the surface of the attracting sphere is approached, and decreases as you recede from it.

To illustrate the effect that should be produced by the operation of these two laws, and also to show how such required effect is modified by the operation of the equilibric force, we make use of the figures 1 and 2 :

Fig. 1. Fig. 2.

In the Figures 1 and 2, *A* and $A^1$ represent a bar of metal, say one inch square and five inches in length, that assumes a position horizontal to the surface of the earth *B*, when in a state of rest, and supported at a point equidistant from its ends on the knife edge fulcrums *C* and $C^1$.

In Fig. 1, the two ends of the bar *A*, at *d* and *e*, are equidistant from the surface of the earth at *B*, and all parts of the bar are subject to an equal gravitative attraction, and its weight and length are equally distributed upon either side of its fulcrum *C*.

Now, if by a gentle blow on the top of the bar at *d*, it be started downward towards the earth *B*, the momentum of its two ends will be in the line of a circle around its fulcrum in the direction indicated by the arrows, the momentum of all parts of the bar acting in unison, to make it revolve upon its fulcrum as its center of motion.

According to the law as above stated, the gravitative force exerts an increasing attraction upon the decending end at *d*, and a decreasing attraction upon the ascending end at *e*, and thus we find that both the momentum of the bar and the attractive force of gravitation, are acting in unison to continue the motion imparted to the bar by the blow that disturbed its equilibrium, and changed its condition from a state of rest to a state of motion, and according to these laws, it should continue to move until it assumed the perpendicular position at least, and it is difficult to discover why, if suspended upon a pivot and placed in a vacuum, it should not continue to revolve upon its center from the effect of momentum and gravity.

Yet, notwithstanding the co-operative effort of momentum and gravitation to perpetuate its motion, we find that it comes to a state of rest in the position shown in Fig. 2.

Let us examine it while in this position of temporary rest. We find that the distance from *f* to *h* represents $\frac{7}{12}$ of the length of the bar $A^1$, and from *h* to *g* but $\frac{5}{12}$, thus $\frac{7}{12}$ of its weight is upon the left hand side of its supporting fulcrum. But this is not all of the anomaly, for it also appears that the relative distances of *f* and *e*, from the dotted line *h*, *i*, are as 7 to 5, thus in addition to the weight upon the left side of the fulcrum being as 7 to 5, the leverage also is as 7 to 5.

We now perceive that the force of equilibrium has not only to overcome the force of momentum, assisted in some degree by gravitation, but also the co-operation of weight and leverage, that at the point at which they are to be overcome attained to a ratio of 7 to 5.

This, then, is the condition of the bar $A^1$ as represented in Fig. 2. It is in a state of rest, with $\frac{7}{12}$ of its weight, and $\frac{7}{12}$ of its length to the left side of its sustaining fulcrum, and its left end nearer by twice its diameter, to the point of greatest gravitative force in the plane of the surface of the earth at *B*.

The equilibric force acting upon it at this point and in this state, not only overcomes the adverse difference in weight, leverage and gravitation, but also its inertia, and acts as a compelling force until it has carried it to the position shown in Fig. 1, and then as a retarding force, preventing the end *e* from descending as far below the horizontal plane as did the end *d*, and thus alternating its action it finally brings the bar to a state of rest in the true plane of its equilibrium.

We thus demonstrate that equilibrium is the dominating mode of force, and therefore, that the statement that "*A body once put in motion will always continue to move until stopped by the action of a resisting medium,*" is not the statement of a *fact*, unless the equilibric force be called a resisting medium.

## Figures and Tables

**Fig. 1. f1:**
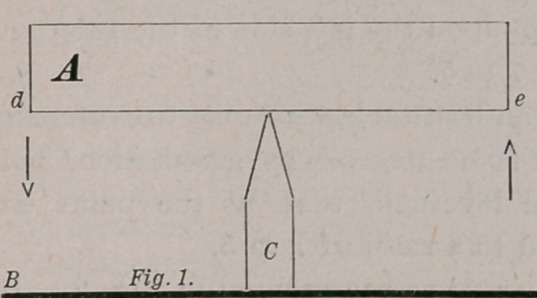


**Fig. 2. f2:**